# Modulation of NK Cell Function by Genetically Coupled C-Type Lectin-Like Receptor/Ligand Pairs Encoded in the Human Natural Killer Gene Complex

**DOI:** 10.3389/fimmu.2013.00362

**Published:** 2013-11-07

**Authors:** Yvonne Bartel, Björn Bauer, Alexander Steinle

**Affiliations:** ^1^Institute for Molecular Medicine, Goethe-University Frankfurt am Main, Frankfurt am Main, Germany

**Keywords:** natural killer gene complex, C-type lectin-like receptors, NK cell receptors, immunomodulatory, cytolysis

## Abstract

Functional responses of natural killer (NK) cells including eradication of “harmful” cells and modulation of immune responses are regulated by a broad variety of activating and inhibitory NK receptors. Whereas the leukocyte receptor complex (LRC) encodes for NK receptors of the immunoglobulin superfamily, genes of C-type lectin-like NK receptors are clustered in the mammalian natural killer gene complex (NKC). Besides the thoroughly studied C-type lectin-like receptors NKG2D, CD94/NKG2x, and members of the murine Ly49 subfamily, the NKC also encodes for NK receptors of the less characterized NKRP1 subfamily. The prototypic mouse NKRP1 receptor is Nkrp1c (also known as NK1.1), while human members of the NKRP1 subfamily are NKRP1A, NKp80, and NKp65. The latter are not straight homologs of mouse NKRP1 receptors, but share distinct subfamily-specific traits classifying them as members of the NKRP1 subfamily. Ligands of the human NKPR1 receptors are likewise C-type lectin-like glycoproteins belonging to the CLEC2 subfamily (i.e., LLT1, AICL, and KACL), and are encoded in the NKC in tight genetic linkage to their respective receptors. Similarly, certain members of the mouse NKRP1 subfamily interact with genetically coupled CLEC2 glycoproteins, while the reasons for this intriguing tight genetic linkage remain unknown. Recent studies provided new and unique insights into the expression, interaction, and signaling of NKRP1 receptors and their ligands, thereby substantially advancing our understanding of their function and biology. Here, we review our current knowledge on NKRP1 receptors and their genetically linked CLEC2 ligands with an emphasis on the human receptor/ligand pairs NKRP1A-LLT1, NKp80-AICL, and NKp65-KACL.

## Structure and Function of NK Cell Receptors

Natural killer (NK) cells perform diverse functions: they not only eradicate virus-infected, malignantly transformed, or stressed cells by virtue of their cytotoxic capabilities, but also produce chemokines and cytokines such as IFNγ, thereby modulating immune responses and contributing to tissue homeostasis (1–4). To serve this variety of purposes, NK cells express a plethora of germline-encoded activating and inhibitory receptors that, in concert, regulate their activities (5–7).

There are two main structural classes of NK receptors with the respective genes clustered at two distinct sites in the mammalian genome: NK cell receptors of the C-type lectin-like superfamily, which will be discussed in detail in this review, are encoded in the natural killer gene complex (NKC; human chromosome 12), whereas the leukocyte receptor complex (LRC; human chromosome 19) codes for immunoglobulin (Ig)-like NK cell receptors (8, 9). Inhibitory NK receptors ligating classical major histocompatibility complex (MHC) class I molecules are either LRC-encoded [e.g., killer cell Ig-like receptors (KIR) in humans] or NKC-encoded (e.g., C-type lectin-like Ly49 receptors in rodents) depending on the respective mammalian order (8, 10). These molecular MHC class I sensors allow the release of NK cell cytotoxicity toward MHC class I-deficient cells typically arising upon viral infection or during tumor formation, and, hence, represent the molecular substrates of the “missing-self” recognition mode (10–12). In contrast, most activating NK cell receptors are conserved among mammalians such as the LRC-encoded Ig-like natural cytotoxicity receptor (NCR) NKp46 and the NKC-encoded, ligand-promiscuous C-type lectin-like receptor (CTLR) NKG2D (9, 13, 14), that both enable molecular recognition of malignant or infected cells by NK cells. In addition to NKG2D and Ly49 receptors, the mammalian NKC encodes for several dozens of other CTLR expressed by various types of hematopoietic cells including NK cells, with some of these CTLR still being poorly explored (9).

## C-Type Lectin-Like NK Receptors Encoded in the Natural Killer Gene Complex

The immune-related CTLR of the NKC have been classified as CTLR of the subgroup V within the realm of multiform C-type lectins (15, 16). This subgroup comprises atypical C-type lectins that have lost the ability to bind carbohydrates via Ca^2+^ complexation and instead interact with proteins as their natural ligands (9, 16). They are type II transmembrane glycoproteins with an aminoterminal cytoplasmic domain, a single transmembrane domain followed by a stalk region and a single extracellular C-type lectin-like domain (CTLD) at the carboxyterminus (9, 16). This name-giving CTLD basically is built up by two α-helices and two antiparallel β-sheets creating a compact structure that is stabilized by two or (mostly) three conserved intramolecular disulfide bonds. Another typical feature is the “WIGL” motif, a stretch of four hydrophobic amino acids that forms the core of the CTLD (9, 16). Functional NKC-encoded CTLR usually form homo- or heterodimers disulfide-linked via paired cysteines of the stalk region that – like the cytoplasmic domain – exhibits considerable length variations among NKC-encoded CTLR.

Natural killer cell-encoded CTLR have been classified based on the expressing cell type either as killer cell lectin-like receptors (KLR) for NK cell-associated CTLR or C-type lectin receptors (CLEC) expressed by non-NK cells.

## NKC-Encoded NK Receptors Bind Either MHC Class I-Like or C-Type Lectin-Like Ligands

**Figure 1:**
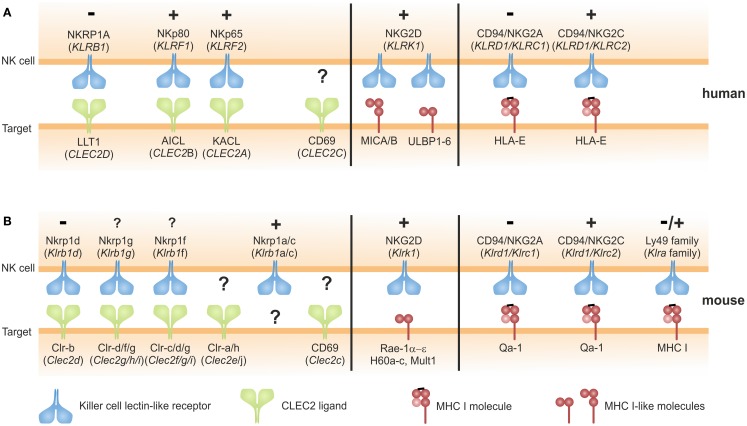
**Ligands of NKC-encoded C-type lectin-like NK cell receptors: C-type lectin fold versus MHC class I fold**. **(A,B)** NK cell receptors encoded in the human **(A)** or in the mouse NKC **(B)** engage two structural types of ligands: ligands of NK cell receptors of the NKRP1 subfamily likewise are C-type lectin-like receptors and members of the CLEC2-subfamily encoded in tight genetic linkage to their respective receptors in the NKC (left). Ligands of Nkrp1a, Nkrp1c, Clr-a, Clr-h, and CD69 remain to be identified. Ligands of CD94/NKG2x receptors and Ly49 receptors (mouse only) are MHC class I complexes consisting of a heavy chain, β2-microglobulin, and a peptide (right). The activating NKG2D receptor binds multiple ligands related to the MHC class I heavy chain not associated with β2-microglobulin or antigenic peptides (middle). Symbols “+” and “−” indicate activating and inhibitory function, respectively, of the respective receptors as reported. For Nkrp1a, Nkrp1f, and Nkrp1g, functional consequences of receptor triggering remain to be addressed.

Most NK cell-associated CTLR (KLR) are known to bind glycoproteins with an MHC class I-like fold: these include classical and non-classical MHC class I molecules, and MHC class I-like molecules (Figure 1). A prominent member of this group is NKG2D (*KLRK1*), an activating receptor that binds to several MHC class I-like molecules induced by various forms of cellular stress such as viral infection, tumor formation, tissue damage, and heat shock (17, 18). Another example are the murine Ly49 receptors detecting allelic variants of MHC I molecules and the CD94/NKG2x receptors interacting with a non-classical MHC class I molecule presenting signal peptides of MHC class I molecules (9, 12) (Figure 1). These NK receptors share their MHC class I ligands with T cell receptors (TCR) of cytotoxic T cells. According to current belief, this molecular partner-sharing between TCR of CD8 T cells and MHC class I-specific inhibitory receptors of NK cells primarily evolved owing to the evolutionary imperative to bail the adaptive immune system out of its MHC-centric single-mindedness, i.e., establishing “missing-self” recognition of NK cells as a safeguard for CD8 T cell-blinding in case of abrogated MHC class I expression.

**Figure 2:**
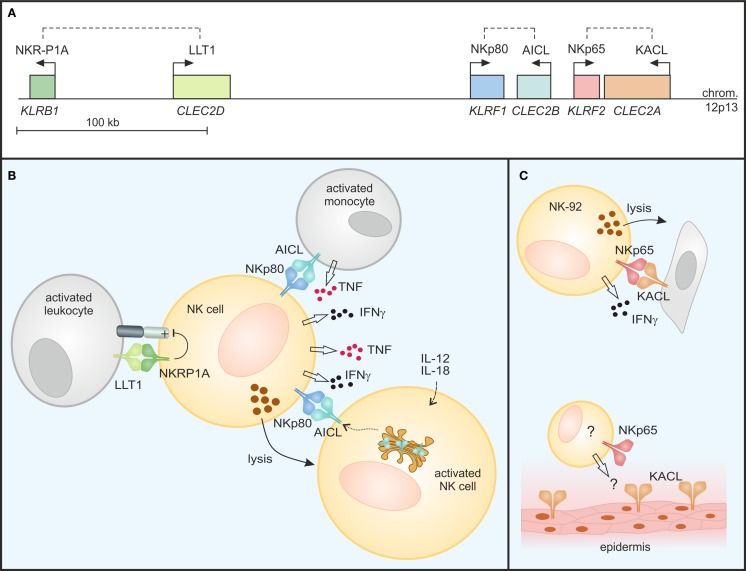
**Modulation of NK cell responses by C-type lectin-like receptor/ligand pairs encoded in the human NKC**. **(A)** NKRP1 receptors and their respective ligands of the CLEC2 subfamily are encoded in tight genetic linkage in the telomeric subregion of the human NKC on chromosome 12. Genes (*italics*) are depicted true to scale with transcriptional orientations indicated by arrows and receptor-ligand pairs by dashed lines. **(B)** NKRP1A inhibits cytotoxicity and IFN-γ secretion of NK cells upon binding of LLT1 expressed on activated leukocytes. In contrast, NKp80 stimulates effector responses of NK cells: NKp80-AICL interaction promotes TNF and IFNγ production in an activating cross-talk between NK cells and monocytes in the presence of inflammatory cytokines. Further, “resting” NK cells also respond to monokine-activated NK cells in an NKp80-dependent manner by cytokine secretion and cytotoxicity: AICL surfaces on activated NK cells from stores in the Golgi complex, rendering these susceptible to NKp80-mediated cytolysis by bystander NK cells. **(C)** NKp65 stimulates cytotoxicity and IFNγ secretion of NK-92 cells upon encounter of KACL-bearing target cells. While KACL is specifically expressed by keratinocytes of the human epidermis, cells endogenously expressing NKp65 remain undefined.

In a distinct subregion of the mammalian NKC, there is a gene cluster of a second class of KLR comprising members of the NKRP1 (NK receptor protein) subfamily. In contrast to other KLR, these NKRP1 receptors do not bind MHC class I-like ligands. The prototypic NKRP1 family member is the mouse activating receptor Nkrp1c (NK1.1) representing one of the earliest reported markers of mouse NK cells (19). While the ligand (and thus the function) of Nkrp1c still remains elusive, other NKRP1 receptors in mouse, rat, and man have been shown to engage structurally highly related CTLR belonging to the CLEC2 subfamily whose genes are interspersed in the NKC among the NKRP1 genes (20, 21) (Figure 1). In mice, most CLEC2 family members are represented by C-type lectin-related (Clr) molecules (Clr-a,-b,-c,-d,-f,-g,-h) (20). Originally, the mouse receptors Nkrp1d and Nkrp1f have been shown to engage the genetically linked Clr molecules Clr-b and Clr-g, respectively, and Nkrp1d/Clr-b interaction has been proposed to represent another form of “missing-self” recognition, as Clr-b expression was often diminished on tumor cell lines (22, 23). Another rather prominent representative of the CLEC2 family is CD69 (*CLEC2C*): CD69 is well-known for its rapid cell-surface appearance on lymphocytes upon activation (24) and has been involved in the retention of activated lymphocytes in lymphoid organs by virtue of a cis interaction with sphingosine-1-phosphate receptor 1 (25). As for Nkrp1c (NK1.1), a trans-acting receptor of CD69 has not yet been identified. CD69 is the only CLEC2 family member conserved both in man and rodents. Other human CLEC2 family members are “keratinocyte-associated C-type lectin” (KACL; encoded by the *CLEC2A* locus), “activation-induced C-type lectin” (AICL; *CLEC2B*), and “lectin-like transcript 1” (LLT1; *CLEC2D*) (Figure 2A) that are equidistant relatives of mouse Clr molecules and share CLEC2-subfamily traits such as the “FLkRy” motif in the α2 helix (for dimerization), a short L3 loop, and a frequent replacement of CTLD cysteines 4 and 5, which are highly conserved in other CTLR (21). These CLEC2 family members are ligands of the human NK receptors NKp80, NKp65, and NKRP1A (or CD161) that do share a couple of unique features as outlined in the following chapters and therefore reasonably can be grouped as human NKRP1 receptors. It should be noted, however, that among these, NKRP1A is most closely related to mouse NKRP1 receptors.

## Genetically Linked NKRP1 Receptor-CLEC2 Ligand Pairs in the Human NKC

The three human CLEC2 glycoproteins LLT1, AICL, and KACL specifically bind to the respective close-by encoded human NKRP1 receptors with the inhibitory CTLR NKRP1A (encoded by the *KLRB1* locus) ligating LLT1, and the activating receptors NKp80 (*KLRF1*) and NKp65 (*KLRF2*) binding to AICL and KACL, respectively (Figures 1 and 2) (26, 27). The reasons for this characteristic tight genetic linkage of NKRP1 receptor-CLEC2 ligand pairs are unknown, but certainly are of considerable interest for a thorough understanding of their functional relevance. Notably, no criss-cross-reactivity has been detected between these human NKRP1 receptors and other CLEC2 family members in binding assays with the respective purified CTLD apart from their above-mentioned dedicated interaction (26). This is different for certain members of the mouse NKRP1 receptors, some of which have been reported to promiscuously bind to several Clr molecules: e.g., Nkrp1f binds to Clr-c, Clr-d, and Clr-g, while Nkrp1g binds to Clr-d, Clr-f, and Clr-g (23, 28, 29). Crucial insights into specificity and promiscuity of NKRP1–CLEC2 interactions can be expected from analyses of crystal structures as described in the following.

## Interaction of Look-Alikes: Structural Aspects of NKRP1–CLEC2 Interaction

Up to date, several crystal structures of NKC-encoded CTLR in complex with MHC class I-like ligands have been solved such as the NKG2D/MICA complex (30), CD94/NKG2A bound to HLA-E (31, 32), and mouse Ly49A in complex with its MHC class I ligand H-2D^d^ (33).

In contrast, the mode of interaction of NKRP1-CLEC2 pairs remained unknown, as only unligated structures of NKRP1 [i.e., Nkrp1a (34)] or CLEC2-subfamily members [i.e., CD69 (35, 36), and Clr-g (37)] have been reported. However, the first structure of a NKRP1-CLEC2 receptor/ligand pair became available very recently showing NKp65 in complex with its ligand KACL, also representing the first complex structure of two interacting CTLD (38).

The complex of NKp65/KACL reveals a KACL homodimer that symmetrically binds two NKp65 monomers in a bivalent binding mode, i.e., one NKp65 monomer is bound by each KACL subunit in an identical manner. Both, NKp65 and KACL are structurally very similar (CTLD sequence identity: 29%) and interact via the membrane-distal surface of the CTLD in a head-to-head orientation. Hence, binding of a KACL subunit to an NKp65 monomer resembles a symmetrical homodimer (38). It has been noted that this symmetrical, butterfly-shaped assembly of the NKp65/KACL complex exhibits some similarities with the Ly49C/H-2K^b^ complex in which the dimeric Ly49C receptor interacts with two MHC class I ligands (39). Contrary to this, other NKC-encoded receptors such as NKG2D, CD94/NKG2A, and Ly49A follow a different binding mode in which a dimeric CTLR engages only a single MHC class I(-like) molecule.

The large interface of the NKp65-KACL complex is mainly hydrophobic, contains numerous hydrogen bonds and stands out due to a high shape complementarity (38). Collectively, these features explain the exceptionally high affinity in the low nanomolar range that also may compensate for the monomeric state of NKp65 (38). Differently from the two other human NKRP1-CLEC2 receptor-ligand pairs, neither NKp65 nor KACL are disulfide-linked. Originally, homodimerization was proposed for both, NKp65 and KACL (26), but the crystal structure of the NKp65-KACL complex reveals a monomeric NKp65, while confirming homodimerization of KACL (38). The comparison of the structures for KACL, human CD69, and mouse Clr-g shows a very similar dimerization mode of the CTLD with the two subunits primarily interacting through the β0 strand and the α2 helix (35–38).

As the sequence relatedness of the CTLD of KACL, AICL, LLT1, and mouse Clr molecules as well as that of the mouse and human NKRP1 receptors suggest a high structural similarity, the NKp65-KACL structure was proposed to be representative for all genetically linked NKRP1-CLEC2 pairs encoded in the NKC (38). Mariuzza and colleagues define five key binding residues of KACL in the interaction with NKp65 that are either highly conserved or conservatively substituted among all three related human CLEC2 glycoproteins (KACL, AICL, and LLT1) (38). Notably, these amino acids make contact to residues of the respective receptors (NKp65, NKp80, and NKRP1A) which, themselves, are either strictly conserved or conservatively substituted. Consequently, these key binding residues might drive the interaction of NKC-encoded receptor/ligand pairs to adopt a conserved binding topology, while specificity of a receptor for a certain ligand may be determined by additional, less conserved amino acids in the binding interface. Based on structural data or mutational analyses, other previous studies suggested an interaction mode for certain NKRP1-CLEC2 receptor/ligand pairs (i.e., Nkrp1f/Clr-g; NKRP1A-LLT1) primarily based on electrostatic complementarity (37, 40).

## Engagement of LLT1 by NKRP1A Modulates Immune Responses

An early report by Lanier and colleagues characterized the NKRP1A receptor as a human homolog of mouse inhibitory Nkrp1 receptors expressed as a disulfide-linked homodimer on most human NK cells (41). In the following, NKRP1A expression was also reported for NKT cells, various subsets of T cells including Th17 cells, as well as groups of innate lymphocytes (42–46). The seminal report by Yokoyama and colleagues of certain mouse NKRP1 receptors engaging ligands of adjacently encoded Clr molecules (23) also stimulated the research on ligands of human NKRP1 receptors resulting in the identification of LLT1 as the ligand of NKRP1A (47, 48). LLT1 is the product of one out of several alternatively spliced transcripts of the *CLEC2D* locus, and is primarily expressed on activated lymphocytes and antigen presenting cells such as Toll-like receptor (TLR)- or B cell receptor (BCR)-activated B cells (49–51). LLT1 expression on antigen presenting cells is enhanced by IFNγ and inducible on B cells by infection with HIV or Epstein–Barr virus, as well as in inflamed tonsils (50). When bound by LLT1, NKRP1A inhibits cytotoxicity and IFNγ production of NK cells thus impairing NK cell responses toward B cells (Figure 2B) (47, 48). Expression of LLT1 on TLR-stimulated plasmacytoid and monocyte-derived dendritic cells (DC) might, at least in parts, explain the resistance of mature DC toward NK cell-mediated cytolysis (51).

Overall, NKRP1A-LLT1 interaction may contribute together with the MHC class I-specific inhibitory receptors and CD94/NKG2A to NK self-tolerance. Along these lines, it has been proposed based on studies with glioblastoma, that aberrant expression of LLT1 is exploited by malignant cells to avert NK cell-mediated tumor elimination (52).

While NKRP1A clearly functions as an inhibitory receptor on NK cells, the role of NKRP1A on T cells appears ambiguous as evident from a series of studies by various laboratories: NKRP1A engagement has been reported to costimulate T cell proliferation and cytokine secretion by activated T cells (47, 50, 53), to provide costimulation for NKT cells (42), but also to reduce release of TNF by CD8 T cells (51). Obviously, NKRP1A ligation differentially impacts on NK and T cell function *in vitro*, while the immunological significance of NKRP1A engagement by LLT1 for the various NKRP1A-expressing lymphocytes *in vivo* remains poorly understood. Studies on NKRP1A signaling cascades in NK versus T cells, as well as *in vivo* studies with humanized mice may be suitable to further our understanding of NKRP1A function.

## Immunomodulatory Functions of NKp80-AICL Interaction

The disulfide-linked homodimeric CTLR NKp80 originally was identified by Moretta and colleagues as an activating receptor rather specifically expressed by human NK cells (54). Subsequent studies showed that NKp80 is conserved among primates but absent from rodents (55, 56) and also present on certain γδ T cells as well as a subset of effector memory CD8 αβ T cells that are characterized by high cytotoxic responsiveness and an inflammatory NK-like phenotype (27, 57). In contrast to the expression on virtually all human NK cells, NKp80 expression is absent from human NK cell lines (58).

Activation-induced C-type lectin was uncovered as an NKp80 ligand when pursuing the hypothesis that receptor and ligand may be encoded in genetic linkage (27). Indeed, the genes of NKp80 and AICL are located in a tail-to-tail orientation only 7 kb apart from each other (27) (Figure 2A). An earlier report on the induction of AICL transcripts upon activation of peripheral blood mononuclear cells led to the term AICL (59). Later, expression of AICL glycoproteins was observed for myeloid cells, including macrophages, granulocytes, and TLR-stimulated monocytes, while differentiation of monocytes to DC is accompanied by a concomitant decrease in AICL expression (60).

Functional studies characterized NKp80 as an activating NK cell receptor triggering cytotoxicity and promoting the release of the proinflammatory cytokines IFNγ and TNF (27, 54). AICL is expressed on some human myeloid cell lines, most prominently on U937 (27, 54), and expression by a few non-hematopoietic cell lines and primary human liver cancer cells has also been reported (61). Accordingly, NKp80-AICL interaction stimulates cytolysis of malignant AICL-expressing myeloid cells by NK cells and effector memory CD8 T cells (27, 57). These findings raise the possibility that NKp80-AICL interaction may contribute to NK-mediated surveillance of myeloid leukemia cells. Notably, cells infected with Kaposi’s sarcoma-associated herpes virus (KSHV) have been shown to downregulate AICL through the action of a viral ubiquitin ligase, thereby providing protection against NKp80-mediated cytotoxicity (62). Possibly, a similar mechanism of AICL retention or downregulation may also be exploited by malignant cells.

NKp80-AICL interaction not only promotes NK cell-mediated cytolysis of malignant myeloid cells, but also is critically involved in the mutual activating cross-talk between NK cells and monocytes under inflammatory conditions stimulating secretion of IFNγ and TNF, respectively (27, 63) (Figure 2B). Similarly, contribution of NKp80 to the mutual activation of effector T cells and macrophages under inflammatory conditions was observed (57). Hence, NKp80-AICL interaction may be involved in the induction and modulation of immune responses during the early phase of infection or maintenance of immune responses during chronic inflammation.

Only very recently, AICL expression by primary human NK cells was reported (58). In resting NK cells, AICL glycoproteins are retained in the Golgi complex primarily due to unresolved interactions of its CTLD. Intracellular retention has also been observed for CD69 expressed by resting lymphocytes while cellular activation led to a rapid mobilization of CD69 to the cell surface (64). Similarly, AICL surfaces on NK cells activated upon PMA-treatment or exposure to monokines IL-12 and IL-18 (58). Monokine-induced AICL expression on human NK cells is paralleled by downregulation of NKp80 leading to a loss of NKp80-mediated responsiveness. Instead, AICL expression on monokine-activated NK cells enabled functional recognition by autologous “resting” NK cells in an NKp80-dependent manner stimulating cytokine production and cytotoxicity of the latter (Figure 2B). As memory-like properties of NK cells are induced by exposure to monokines IL-12 and IL-18 (65), e.g., in secondary lymphoid organs and at sites of inflammation, one might hypothesize that upregulation of AICL and subsequent NKp80-mediated elimination of monokine-activated NK cells by bystander NK cells contributes to the regulation of the pool of “memory” NK cells. Activated NK cells have previously also been shown to upregulate LLT1 at the cell surface (50) and, hence, a simultaneous engagement of LLT1 by NKRP1A may diminish the activating signals emanating from NKp80 engagement through AICL on activated NK cells.

Of note, human NK cells not only express NKp80 but also the NKp80 ligand AICL, thereby allowing for an autonomous control of human NK cell responses. The observed co-expression of AICL and its receptor NKp80 by the same cell type sheds new light on the tight genetic linkage of NKC-encoded NKRP1/CLEC2 receptor/ligand pairs that might allow for an interdependent regulation of gene expression at the transcriptional level. Limitation of cellular immune responses by NK cells has also previously been reported for NKG2D-mediated recognition and cytolysis of activated T cells (66) and, along these lines, it is of interest whether AICL also is upregulated on activated T cells and B cells.

## KACL and Its Activating Receptor NKp65 Facilitate Immunosurveillance of Keratinocytes

The CTLR encoded by the *CLEC2A* gene was termed KACL (for keratinocyte-associated C-type lectin) due to its almost exclusive expression by human keratinocytes (67). This rather skin-specific expression of KACL is unique among human and mouse CLEC2 family members: several of these are commonly expressed by hematopoietic cells (e.g., LLT1, AICL, CD69, Clr-g), while others exhibit either a very broad (e.g., Clr-b) or a highly restricted expression by epithelial or neuronal tissues (e.g., Clr-f, BACL) (21, 68, 69). Specific expression of KACL by keratinocytes may be indicative for a dedicated role in skin immunobiology, but such a role remains to be shown in context of expression and function of the KACL receptor NKp65 (see below). As KACL is also prominently expressed by the myeloid cell line U937 and residual KACL transcripts were detected in bone marrow, expression by a scarce subset of hematopoietic cells cannot be excluded (67).

Analogous to the genetic linkage of the two related human NKRP1-CLEC2 pairs NKRP1A-LLT1 and NKp80-AICL, the *KLRF2* gene coding for the KACL receptor NKp65 is situated in close proximity to *CLEC2A* in the NKC with both genes only 3 kb apart in a tail-to-tail orientation (26) (Figure 2A). As mentioned earlier, NKp65 binds to KACL with very high affinity (*K*_D_ ∼ 1–10 nM) (26, 38). Hence, the NKp65-KACL interaction is among the strongest known between cell-bound receptors, and considerably stronger than NKp80-AICL (*K*_D_ ∼ 4 μM) and NKRP1A-LLT1 (*K*_D_ ∼ 48 μM) interactions (26, 27, 38, 40). Functional engagement of NKp65 by KACL-expressing transfectants or keratinocytes stimulates effector responses such as cytotoxicity and IFNγ release by the human NK cell line NK-92 (26) (Figure 2C) showing that NKp65 is an activating receptor that facilitates immunosurveillance of human keratinocytes. However, significant expression of NKp65 has so far only been detected for NK-92 cells, while peripheral blood NK cells only contain trace amounts of NKp65 transcripts (26, 38). Hence, cells physiologically expressing NKp65 remain to be identified in order to attribute functional relevance to the NKp65-KACL system and to address a potential involvement in diseases of the skin such psoriasis or even in wound healing.

## Mechanisms of Signaling by Human NKRP1 Receptors

Most NK receptors transduce signals through a couple of tyrosine-based sequence motives with the tyrosine being phosphorylated upon ligand engagement and acting as a starting point of the emerging signaling cascade. The widespread immunoreceptor tyrosine-based activating motif (ITAM) consists of a tandem tyrosine unit with two YxxL modules separated by six to eight amino acids and ITAM-based signaling is a common mechanism in activation of T cells, B cells, and NK cells (7, 70). Usually, ITAM are part of specialized signaling adaptors, variably associating with lymphocyte receptors, thus secluding ligand engagement from signal transduction. In contrast, an activating signaling motif containing only one such tyrosine module, thus termed hemITAM, has been described for the cytoplasmic domain of only a few, mostly myeloid CTLR with ligand binding and initiation of signaling being executed by the identical polypeptide chain (71, 72). The same is true for inhibitory receptors containing immunoreceptor tyrosine-based inhibitory motives (ITIM) in their respective cytoplasmic domains: these ITIM relay inhibitory signals through recruitment of phosphatases that finally dampen or abrogate activating signals (7, 70).

Some activating C-type lectin-like NK cell receptors such as Ly49H and CD94/NKG2C associate with the ITAM-bearing adapter molecule DNAX-activating protein of 12 kDa (DAP12) through charged interactions in their transmembrane domains (5, 73). Other ITAM-bearing adaptor molecules employed by NK cells are CD3ζ or FcϵRIγ associating with the NCR NKp30 and NKp46, the low-affinity Fc receptor CD16, or Nkrp1c (NK1.1) (5, 73). ITAM signaling in NK cells involves phosphorylation by Src family kinases and subsequent recruitment and activation of tyrosine kinases Syk and ZAP-70 that, in turn, signal downstream via phosphatidylinositol-3-kinase (PI3K), phospholipase Cγ (PLCγ), and Vav family members (5, 73). Of note, the CTLR NKG2D almost uniquely associates with DAP10 that contains a YINM motif, triggering cytotoxicity in a Syk-independent mechanism through recruitment of PI3K and a Grb2-Vav1 complex (73, 74).

**Figure 3:**
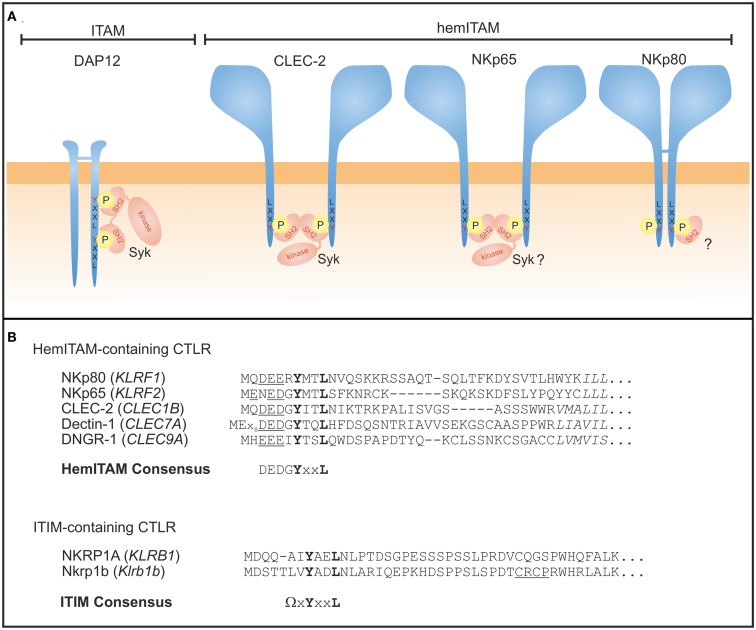
**Signaling modules of human NKRP1 receptors**. **(A)** ITAM-bearing adaptors such as DAP12 are tyrosine phosphorylated upon ligand binding of the associated receptor chain and subsequently recruit Syk kinases via their SH2 domains (left). CLEC-2 is a prototypical hemITAM-containing CTLR expressed by myeloid cells where two juxtaposed and phosphorylated hemITAM are thought to recruit Syk kinase, thereby bridging two adjacent CLEC-2 monomers. The aminoterminal sequences of both, NKp65 and NKp80, comprise a hemITAM-like sequence with tyrosine 7 being essential for signaling. However, Syk binding to NKp65 remains to be shown and recent studies with NKp80 suggest preferred recruitment of a yet unknown signaling protein different from Syk. **(B)** Sequence alignment of hemITAM-containing human activating CTLR expressed by NK cells (NKp80), myeloid cells (CLEC-2, Dectin-1, DNGR-1), or yet unknown cells (NKp65), and of ITIM-containing receptors NKRP1A (human) and Nkrp1b (mouse). The core motif **Y**xx**L** of both hemITAM and ITIM is bolded, the hemITAM-preceding triacidic amino acid sequence underlined, and the first amino acids of the transmembrane domains are in italics. Sequence gaps introduced for alignment are denoted by dashes. HemITAM and ITIM consensus (Ω = L/V/I/S) sequences are also given.

Signaling is rather different for the human activating NKRP1 receptors NKp80 and NKp65 that do not associate with ITAM-containing signaling adaptors such as DAP12. Instead, the cytoplasmic domain of both, NKp80 and NKp65, contains a hemITAM (26, 75) initially described for myeloid-specific CTLR such as CLEC-2 and Dectin-1 (72, 76) (Figure 3). The aminoterminal hemITAM of these CTLR variably matches the consensus sequence DGYxxL with the phosphorylatable tyrosine located at position 7 in all receptors except for Dectin-1 (72). The hemITAM is also consistently situated at a certain distance to the plasma membrane in all CTLR for yet unknown reasons. While the hemITAM-bearing CTLR in myeloid cells stimulate phagocytosis and cytokine secretion, ligation of NKp80 and NKp65 triggers cytotoxic responses by the NK-92 cell line in a hemITAM-dependent manner (26, 75). The hemITAM of CLEC-2 and Dectin-1 have both been shown to recruit and transduce signals through Syk (77–79). With the sequence ERYxxL, the hemITAM of NKp80 significantly differs from the consensus and therefore was classified as an “anomalous hemITAM” (75). Arginine 6 was shown to contribute to a less effective phosphorylation of NKp80, while both glutamate 5 and arginine 6 impaired Syk recruitment resulting in a dampened NKp80-mediated responsiveness (80). The latter could be enhanced by reconstitution of the consensus hemITAM sequence or Syk overexpression (80). As glutamate 5 and arginine 6 are largely conserved among mammalian NKp80 sequences, it has been speculated that dampening of the NKp80-mediated NK cell activation of NK cells evolved to mitigate cytotoxic NK cell responses toward AICL-expressing leukocytes (80).

Syk binding to hemITAM sequences has been studied intensely for CLEC-2 which is only active as a non-disulfide-linked homodimer (81). The molecular mechanisms of CLEC-2 activation seem to be different from that described for ITAM activation since initial phosphorylation of CLEC-2 rather depends on Syk kinase activity than on Src family kinases (82, 83). Thereby, Syk is reported to cross-link CLEC-2 monomers via its tandem SH2 domains (77). This hemITAM cross-binding mode reflects the lack of a second YxxL module in the same polypeptide chain, and may even represent the evolutionary forerunner of an “outsourced” ITAM signaling by secluded and highly specialized adaptor chains with two coupled YxxL modules. Moreover, the triacidic amino acid sequence (DED motif preceding the YxxL motif in CLEC-2; Figure 3B) was shown to be crucial for Syk-dependent signaling (82). Yet, in NKp80, arginine 6 may attenuate these negative charges and thus modulate NKp80 signaling capacity.

The inhibitory mouse Nkrp1b receptor comprises an ITIM in its cytoplasmic domain which recruits Src homology 2-containing protein tyrosine phosphatase-1 (SHP-1) in a phosphorylation-dependent manner (84), and a CXCP motif associated with Lck recruitment (85). In contrast, Lck recruitment of the human NKRP1A receptor is controversial (42, 86) and an association with phosphatases has not been reported so far. Taking this lack of information and the rather heterogeneous functionality reported for NKRP1A (see above) into account, a thorough elucidation of NKRP1A signaling in NK cells and T cells appears necessary. Of note, human NKRP1A has been shown to stimulate activity of acid sphingomyelinase leading to ceramide production and subsequent activation of Akt (53).

Taken together, the activating receptors NKp80 and NKp65, as compared to other human NK receptors, employ distinct and unique signaling mechanisms to stimulate NK cell responses. These mechanistically distinct signaling processes broaden the versatility of NK cells to differentially respond to diverse activating signals in a context-specific manner. Future work will have to characterize the signaling units interacting with the single tyrosine signaling modules of NKp80, NKp65, and NKRP1A, and the emerging downstream signaling pathways.

## Signaling by CLEC2 Family Members

Several studies also reported signaling capacity for NKC-encoded CLEC2 family members. For example, cross-linking of CD69 was reported to trigger cytotoxicity by NK and T cells (87) and to activate Syk in a Src family kinase-dependent manner, which was indispensable for PLCγ and Vav1 phosphorylation (24, 88). Since CD69 contains no tyrosine in the cytoplasmic domain as well as no charged amino acids in the transmembrane domain for recruitment of signaling adaptors, tyrosine-based signal transduction appears unlikely. However, signals may be generated by serine/threonine phosphorylation as reported for DNAM-1 where protein kinase C-mediated serine phosphorylation was shown to be critical for ligand binding and signaling (89). In T cells, CD69 association with JAK3/STAT5 was reported to control Th17 cell differentiation (90). However, a mechanistic link between JAK/STAT signaling pathways and NK cell cytotoxicity is lacking.

Cross-linking of AICL was also shown to trigger TNF secretion in monocytes activated with LPS in a manner comparable to TREM-1 ligation (27) suggesting signaling potential of AICL. Analogous to human NKRP1 family members, AICL lacks positively charged residues in the transmembrane domain that seems to preclude association with ITAM-containing adaptors. The very short cytoplasmic domain of AICL is composed of only seven amino acids and does not offer an obvious signaling motif apart from a single threonine residue that may be subject to phosphorylation. Consequently, mechanisms relaying signals through AICL remain elusive.

## Concluding Remarks

The NKC of both man and mouse encodes for genetically tightly coupled CTLR/ligand pairs consisting of activating or inhibitory NKRP1 receptors and various CLEC2 ligands with a highly diversified tissue expression. As of today, the NKRP1 and CLEC2 families still comprise a couple of orphan CTLR, including Nkrp1c, Nkrp1a, Clr-a, and CD69, where trans-acting ligands remain to be identified for studies on their functional relevance. These receptor/ligand pairs of mice (i.e., Nkrp1/Clr pairs) and humans (i.e., NKRP1A-LLT1, NKp80-AICL, NKp65-KACL) not only share the genetic linkage, but also other characteristics such as structural similarities of the CTLD (among NKRP1 receptors and CLEC2 ligands, respectively), related immunoreceptor tyrosine-based signaling motifs in the cytoplasmic domains of the NKRP1 receptors (and the lack of these in the CLEC2 ligands), a preferential expression of NKRP1 receptors by NK and/or T cells, and CLEC2 ligands with a highly variegated tissue expression, including highly tissue-specific expression patterns (e.g., KACL, Clr-f).

Referring to the tight genetic linkage, it was originally hypothesized that these NKRP1 receptor-CLEC2 ligand pairs might have evolved from an ancient histocompatibility system independent of the MHC-based immunosurveillance system (23). The more recently recognized tissue-specific expression of some of these CLEC2 ligands adds an interesting and supportive facet to this hypothesis, as certain NKRP1 receptor-CLEC2 ligand pairs may have specifically evolved to survey rapidly renewing and pathogen-exposed epithelial barriers (91). Also, specific immunosurveillance of activated (and proliferating) hematopoietic cells, as emerging for the NKp80-AICL system, may go along the same lines.

Collectively, the present, yet rather basic knowledge on these genetically coupled CTLR receptor/ligand units suggests an involvement for some of these in an immunomodulatory cross-talk between hematopoietic cells whereas others may contribute to epithelia-specific immunosurveillance, a hypothesis that awaits to be tested by future research.

## Conflict of Interest Statement

Alexander Steinle filed patents on NKp80 and NKp65. The other co-authors declare that the research was conducted in the absence of any commercial or financial relationships that could be construed as a potential conflict of interest.
